# Supplementary material for: Ultra-low damping insulating magnetic thin films get perpendicular
Source: Nat Commun. 2018 Aug 22;9:3355. doi: 10.1038/s41467-018-05732-1 (PMC6105659; doi:10.1038/s41467-018-05732-1)
Supplement: Supplementary file 1 — Supplementary informations [file 41467_2018_5732_MOESM1_ESM.pdf]

# Ultra-low damping insulating magnetic thin films get perpendicular

Soumah et al.

## Supplementary Notes 1- Derivation of the magneto-elastic anisotropy

The out-of-plane anisotropy constant $K_U$ is ascribed to be a result of, at least, two contributions: a magneto-elastic anisotropy term induced by strain ($K_{\mathrm{MO}}$) and a term that is due to preferential occupation of Bi atoms of non-equivalent dodecahedral sites of the cubic unit cell. This last term is known as the growth induced anisotropy term $K_{\mathrm{GROWTH}}$. From X-ray characterizations and from the known properties of the thick BiYIG LPE grown films, it is possible to calculate the expected values of $K_{\mathrm{MO}}$ in each doping/substrate combination. We thereafter deduce $K_{\mathrm{GROWTH}}$ from the relation $K_{\mathrm{U}} = K_{\mathrm{MO}} + K_{\mathrm{GROWTH}}$. $\mathrm{K_{MO}}$ is directly proportional to the misfit between the film and the substrate:

$$K_{\mathrm{MO}} = \frac{3}{2} \cdot \frac{E\lambda_{111}(a_{film} - a_{substrate})}{(1-\mu)a_{film}} \tag{1}$$

Where $E$, $\mu$ and $\lambda_{111}$ are respectively the Young modulus, the Poisson coefficient, and the magnetostrictive constant along the (111) direction. Those constant are well established for the bulk[1]: $E$= 2.055.10$^{11}$ J.m$^{-3}$, $\mu$= 0.29. The magnetostriction coefficient $\lambda_{111}$ for the thin film case is slightly higher than that of the bulk and depends upon the Bi rate $x$: $\lambda_{111}(x) = -2.819 \cdot 10^{-6}(1 + 0.75x)$ [2]. The two lattice parameter entering in Equation (1): $a_{\mathrm{film}}$ and $a_{\mathrm{substrate}}$ correspond to the lattice parameter of the relaxed film structure and of the substrate. Under an elastic deformation $a_{\mathrm{film}}$ can be derived with the Poisson coefficient:

$$a_{\mathrm{film}} = a_{\mathrm{substrate}} - \left[\frac{1-\mu_{111}}{1+\mu_{111}}\right]\Delta a^{\perp} \tag{2}$$

Where $\Delta a^{\perp} = 4\sqrt{3}d_{444}^{\mathrm{film}} - a_{\mathrm{substrate}}$. All values for the different target/substrate combinations are displayed in the Table S-1. We note here that a negative (positive) misfit corresponding to a tensile (compressive) strain will favor an out-of-plane (in-plane) magnetic anisotropy which is coherent with what is observed in our samples. To estimate the contribution to the magnetic energy of the magneto elastic anisotropy term we compare it to the demagnetizing field $\mu_0 M_{\mathrm{s}}$ that favors in-plane magnetic anisotropy in thin films. Interestingly the magneto elastic field ($\mu_0 H_{\mathrm{MO}}$) arising from $K_{\mathrm{MO}}$ ($\mu_0 H_{\mathrm{MO}} = \frac{2K_{\mathrm{MO}}}{M_{\mathrm{s}}}$) never exceed 30% of the demagnetizing fields and therefore cannot alone be responsible of the observed PMA. Studies on µm-thick BiYIG films grown by LPE showed that PMA in BiYIG arises due to the growth induced anisotropy term $K_{\mathrm{GROWTH}}$ , this term is positive [3] for the case of Bi substitution . We have inferred $\mu_0 H_{\mathrm{GROWTH}}$ values for all films using $K_{\mathrm{U}}$ constants measured by FMR. The results are summarized for thin films grown on sGGG and GGG in respectively Supplementary Figure 1(a) and (b). One can clearly see that $K_{\mathrm{GROWTH}}$ is strongly substrate dependent and therefore does not depend solely on the Bi content. We conclude that strain plays a role in $Bi^{3+}$ ion ordering within the unit cell.

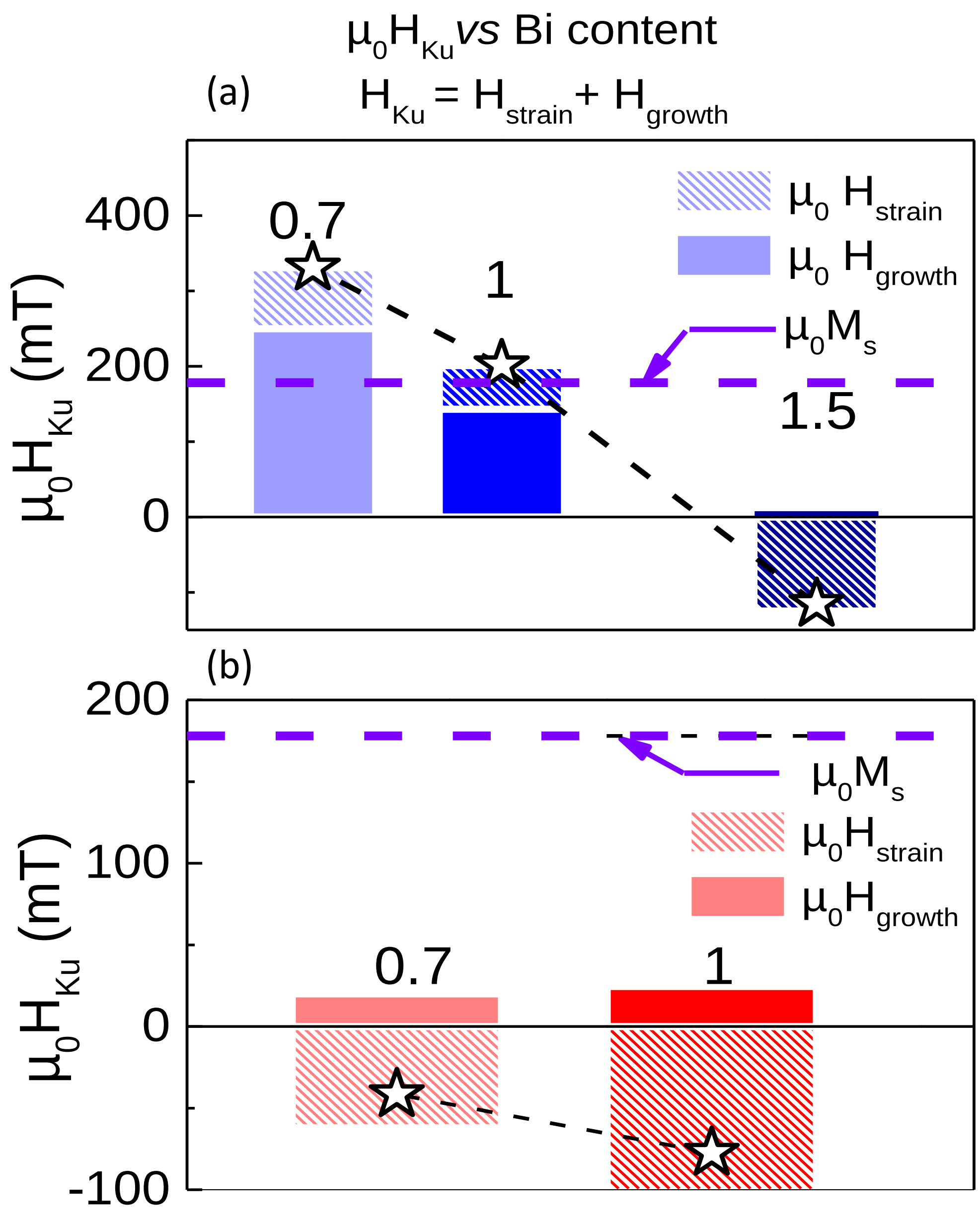


<u>**Supplementary Figure 1**</u>**- Summary of the inferred values of the effective magnetic anisotropy out-of-plan fields for thin films grown on sGGG(a) and on GGG(b).** The horizontal dash lines represent the magnitude of the demagnetization field $\mu_0 M_s$. When $\mu_0 H_{KU}$ is larger than $\mu_0 M_s$ (dot line) films have a PMA, they are in-plane magnetized otherwise.

| Bi content | substrate | $a_{film}$(Å) | $\Delta a^{\perp}/a_{film}$ | $K_{MO}$ ($J.m^{-3}$) | $\mu_0 H_{MO}$(mT) | $\mu_0 H_{demag}$(mT) |
|---|---|---|---|---|---|---|
| 0.7 | sGGG | 12.45 | 0.6 | 5818 | 81 | 179 |
| 0.7 | GGG | 12.41 | -0.4 | -4 223 | -61 | 172 |
| 1.0 | sGGG | 12.47 | 0.3 | 3 958 | 57 | 172 |
| 1.0 | GGG | 12.42 | -0.6 | -6 500 | -102 | 160 |
| 1.5 | sGGG | 12.53 | -0.6 | -8 041 | -124 | 157 |

**Supplementary Table 1- Summary of the films' calculated magneto-elastic anisotropy constant ($K_{MO}$) and the corresponding anisotropy field $H_{MO}$**

## Supplementary Notes 2- Derivation of the domain wall energy

To derive the characteristic domain wall energy $\sigma_{DW}$ for the maze shape like magnetic domains, we use the Kaplan et al. model[4]. This model applies in our case as the ratio of the film thickness ($t_{film}$) to the magnetic domain width ($D_{width}$) is small ($\frac{t_{\mathrm{film}}}{D_{\mathrm{width}}} \sim 0.01$). The domain wall width and the film thickness are then expected to be linked by:

$$D_{width} = t_{film} e^{-\pi 1.33} e^{\frac{\pi D_0}{2t_{film}}} \quad (3)$$

$D_{\mathrm{width}} = t_{\mathrm{film}}\, \mathrm{e}^{-\pi 1.33}\, e^{\frac{\pi D_0}{2t_{\mathrm{film}}}}$ where $D_0 = \frac{2\sigma_{\mathrm{W}}}{\mu_0 M_S^2}$ is the dipolar length. Hence we expect a linear dependence of $\ln\left(\frac{D_{\mathrm{width}}}{t_{\mathrm{film}}}\right)$ vs $\frac{1}{t_{\mathrm{film}}}$ :

$$ln\left(\frac{D_{width}}{t_{film}}\right) = \frac{\pi D_0}{2} \cdot \frac{1}{t_{film}} + Cst \quad (4)$$

The magnetic domain width of $Bi_xY_{3-x}IG$//sGGG ($x$ = 0.7 and 1) for several thicknesses are extracted from 2D Fourier Transform of the Kerr microscopy images at remanence. In Supplementary Figure 2 (a) and (b), we plot $\ln\left(\frac{D_{\mathrm{width}}}{t_{\mathrm{film}}}\right)$ vs $\frac{1}{t_{\mathrm{film}}}$ for $Bi_{0.7}Y_{2.3}IG$//sGGG and $Bi_1Y_2IG$//sGGG respectively. The plot follows the expected linear dependence of Equation (4). We infer from the zero intercept an estimation of the dipolar length $D_0$ of BiYIG films doped at 0.7 and 1 in Bi: $D_0^{x=0.7}$= 16.5 µm and $D_0^{x=1}$= 18.9µm. The corresponding domain wall energy are respectively 0.7 mJ.m$^{-2}$ and 0.65 mJ.m$^{-2}$. Even if the small difference in domain wall energy between the two Bi content may not be significant regarding the statistical fitting errors, it correlates to the decrease of the out-of-plane anisotropy ($K_U$) with increasing the Bi content.

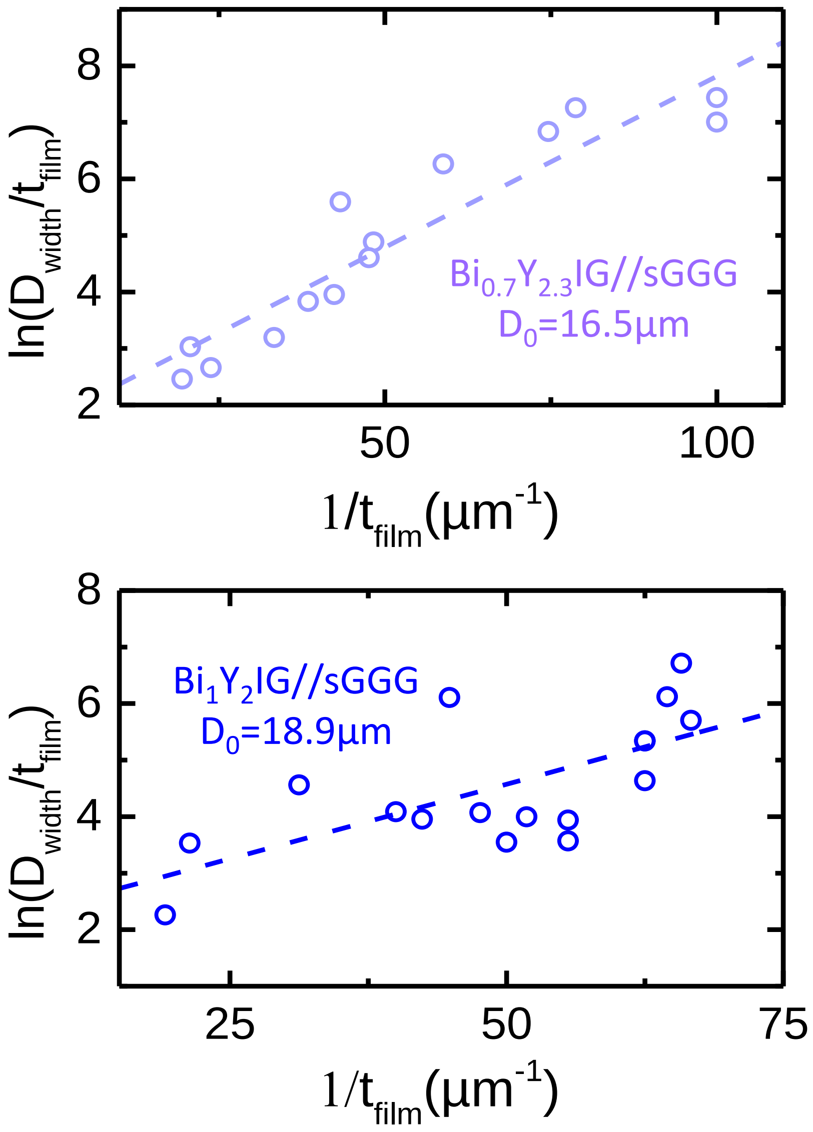


**<u>Supplementary Figure 2</u>- Evolution of the domain width vs film thickness.**

$\ln\left(\frac{D_{\mathrm{width}}}{t_{\mathrm{film}}}\right)$ *vs* $\frac{1}{t_{\mathrm{film}}}$ for $Bi_xY_{3-x}$IG//sGGG films doped at 0.7(a) and 1 (b) in Bi. Dots correspond to the experimental values. The dashed line is the linear fit that allows to extract the $D_0$ parameter.

## Supplementary Notes 3- Damping and effective magnetic field derivation

From In Plane frequency dependent of FMR we can derive the effective magnetization ($M_{\mathrm{eff}}$) using the Kittel law:

$$f_{\mathrm{res}} = \mu_0 \gamma \sqrt{H_{\mathrm{res}}(H_{\mathrm{res}} + M_{\mathrm{eff}})} \tag{5}$$

Where $\gamma$ is the gyromagnetic ratio of the BiYIG (assumed to be same as the one of the YIG): $\gamma$=28 GHz.T$^{-1}$. $H_{\mathrm{res}}$ and $f_{\mathrm{res}}$ are respectively the FMR resonant field and frequency. The uniaxial magnetic anisotropy can thereafter be derived using the saturation magnetization form squid magnetometry using: $M_{\mathrm{eff}}=M_s-H_{\mathrm{KU}}$. The Gilbert damping ($\alpha$) and the inhomogeneous linewidth ($\Delta H_0$) which are the two paramaters defining the magnetic relaxation are obtained from the evolution of the peak to peak linewidth ($\Delta H_{\mathrm{pp}}$) vs the resonant frequency ($f_{\mathrm{res}}$):

$$\Delta H_{\mathrm{pp}} = \Delta H_0 + \frac{\frac{2}{\sqrt{3}} \alpha f_{\mathrm{res}}}{\mu_0 \gamma} \tag{6}$$

The first term is frequency independent and often attributed magnetic inhomogeneity's (anisotropy, magnetization).

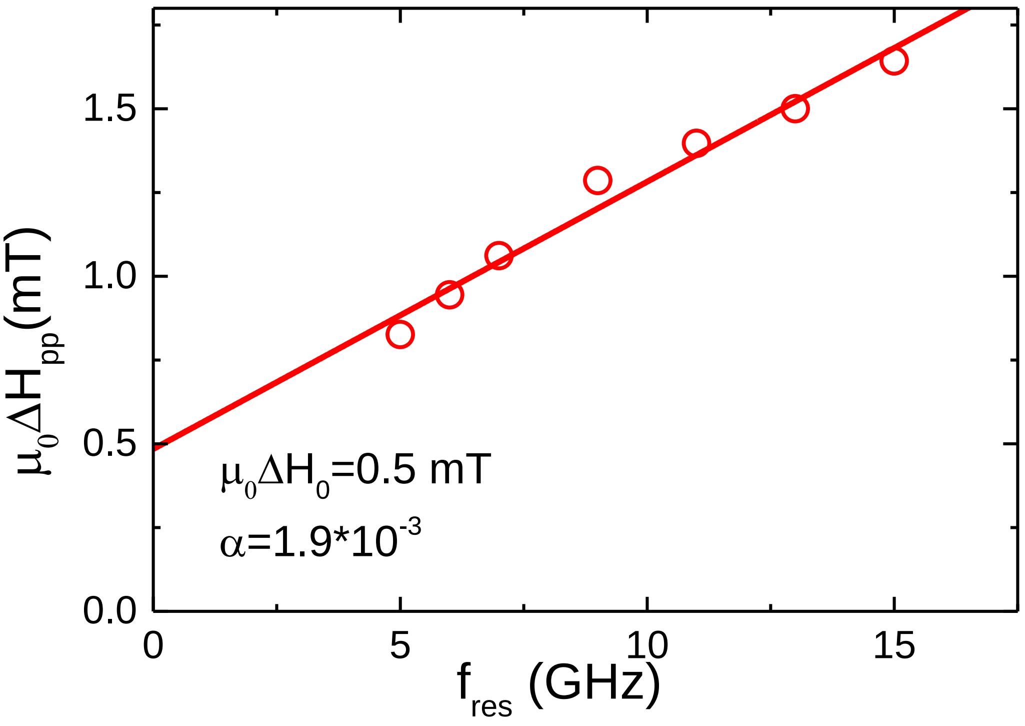


<u>**Supplementary Figure 3**</u>**- $\mu_0\Delta H_{pp}$ vs $f_{res}$ on 18 nm thick $Bi_{15.}Y_{1.5}$IG//sGGG**

The linewidth frequency dependence from 5 to 19 GHz for in plane magnetized $Bi_{1.5}Y_{1.5}$IG//sGGG sample allow to extract the damping and the inhomogeneous linewidth parameter using the Equation (6).

## Supplementary References:


1. Hansen, P., Klages, C. -P. & Witter, K. Magnetic and magneto-optic properties of praseodymium- and bismuth-substituted yttrium iron garnet films. *J. Appl. Phys.* **60,** 721–727 (1986).

2. Ben Youssef, J., , Legall, H. & . U. P. et M. C. Characterisation and physical study of bismuth substituted thin garnet films grown by liquid phase epitaxy (LPE). (1989).

3. Fratello, V. J., Slusky, S. E. G., Brandle, C. D. & Norelli, M. P. Growth-induced anisotropy in bismuth: Rare-earth iron garnets. *J. Appl. Phys.* **60,** 2488–2497 (1986).

4. Kaplan, B. & Gehring, G. A. The domain structure in ultrathin magnetic films. *J. Magn. Magn. Mater.* **128,** 111–116 (1993).
